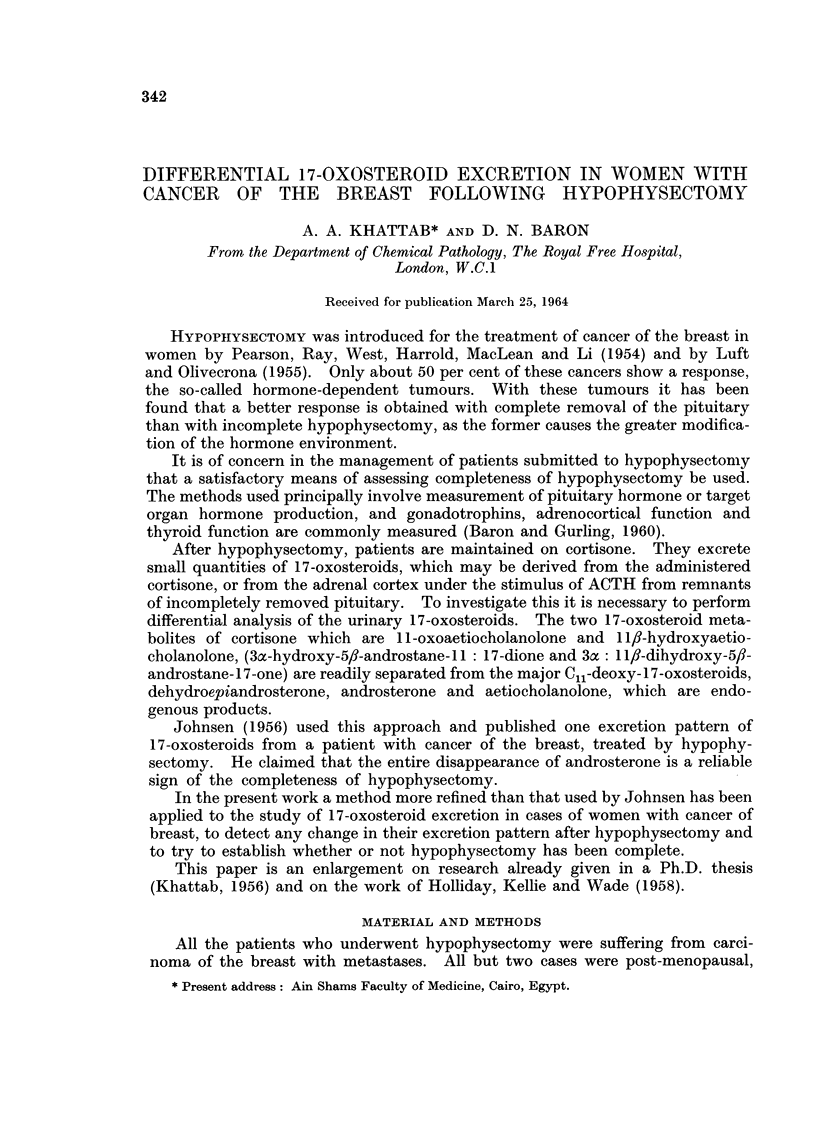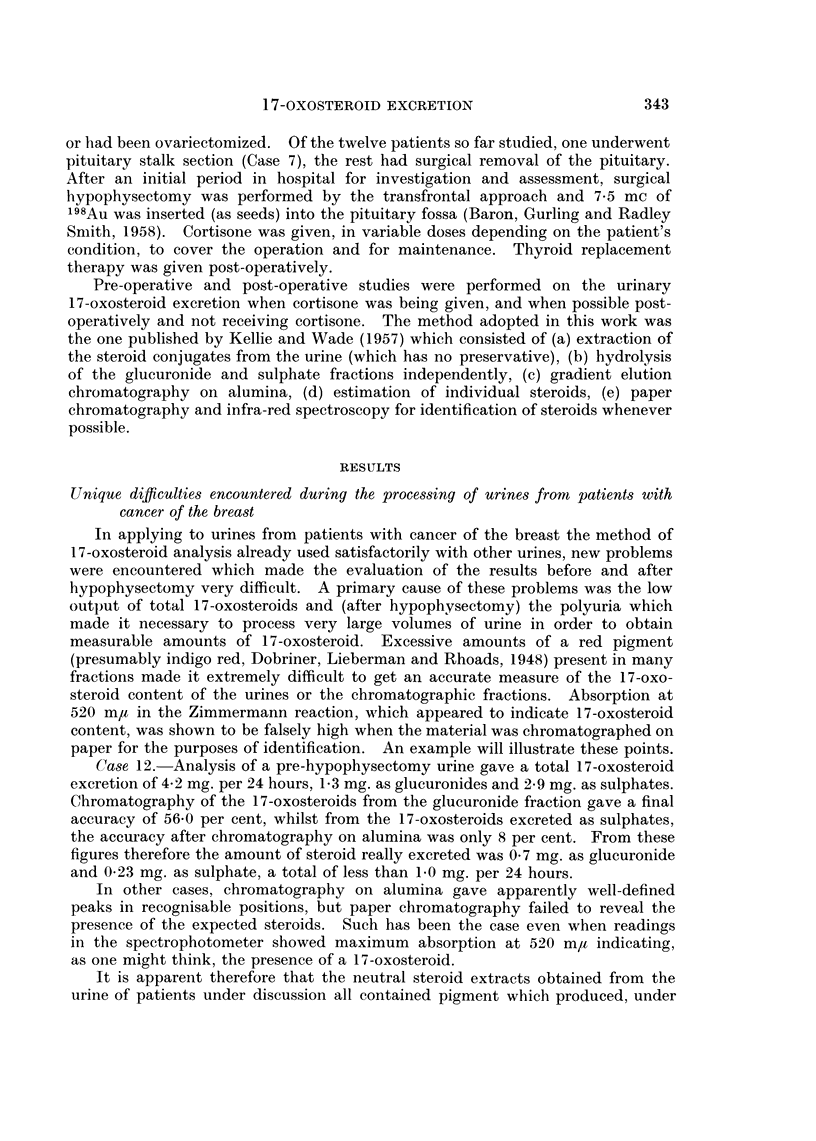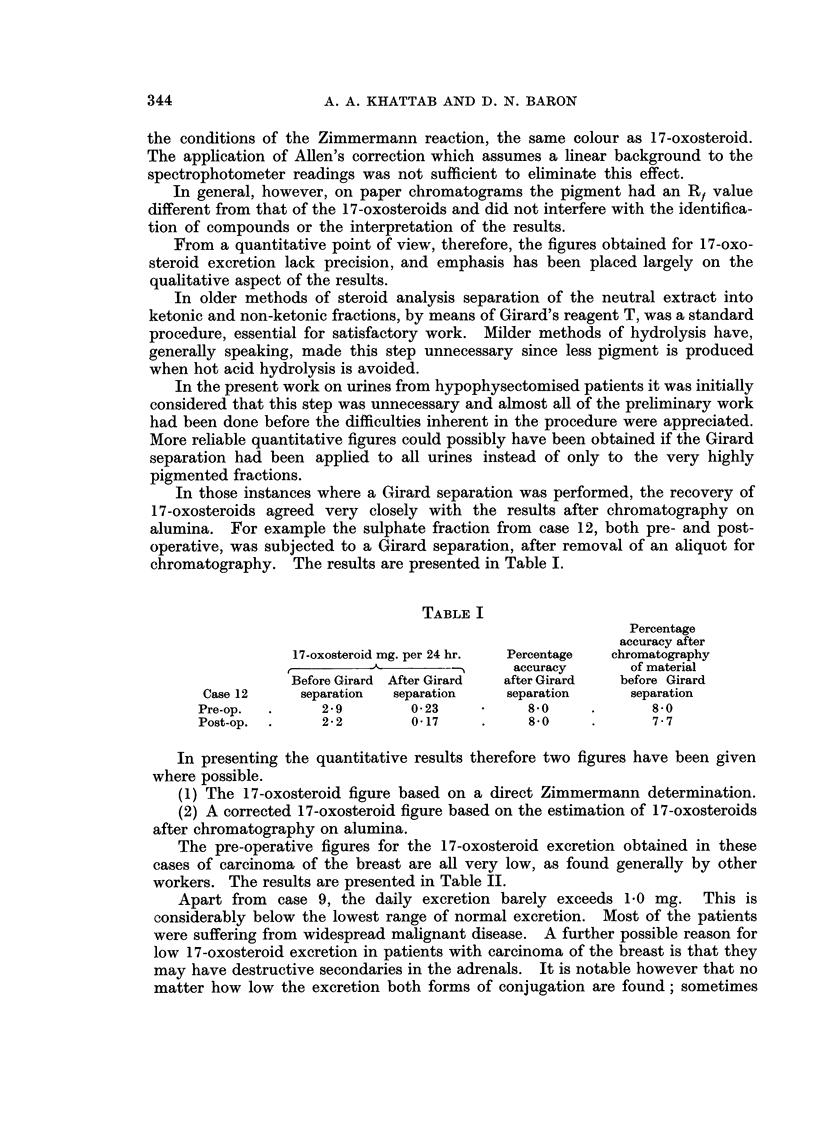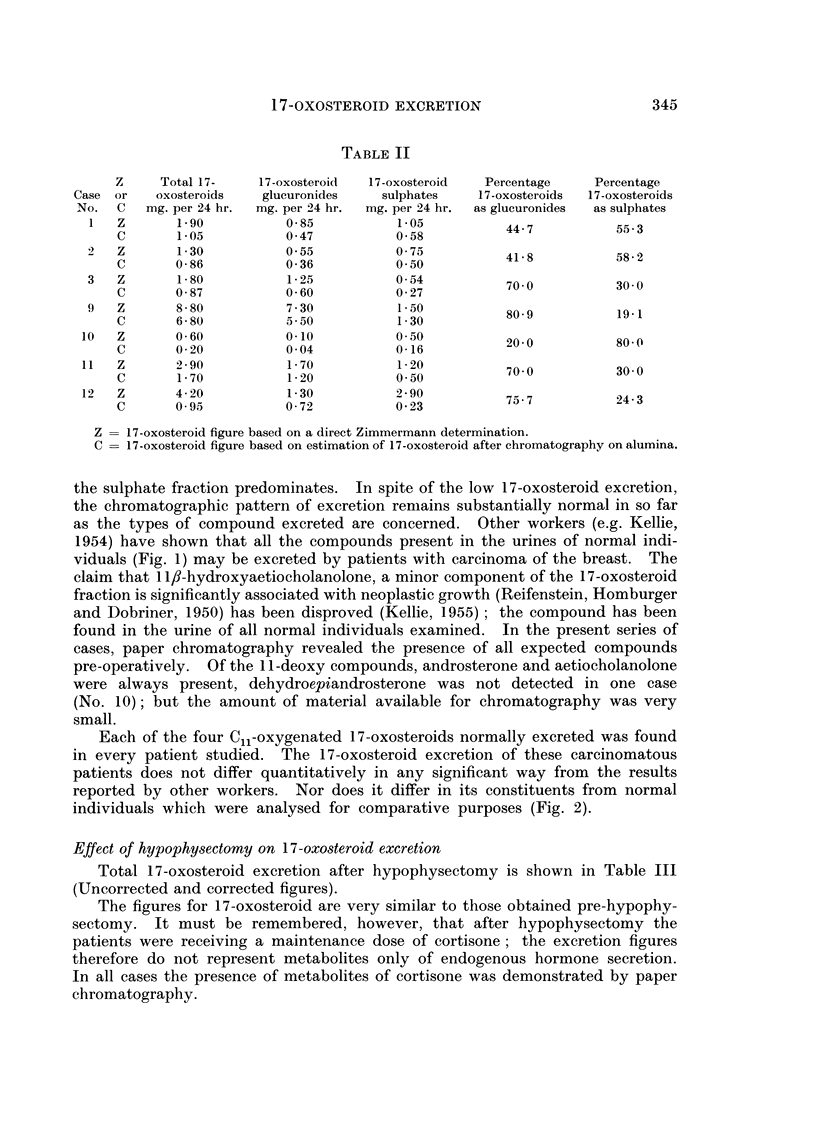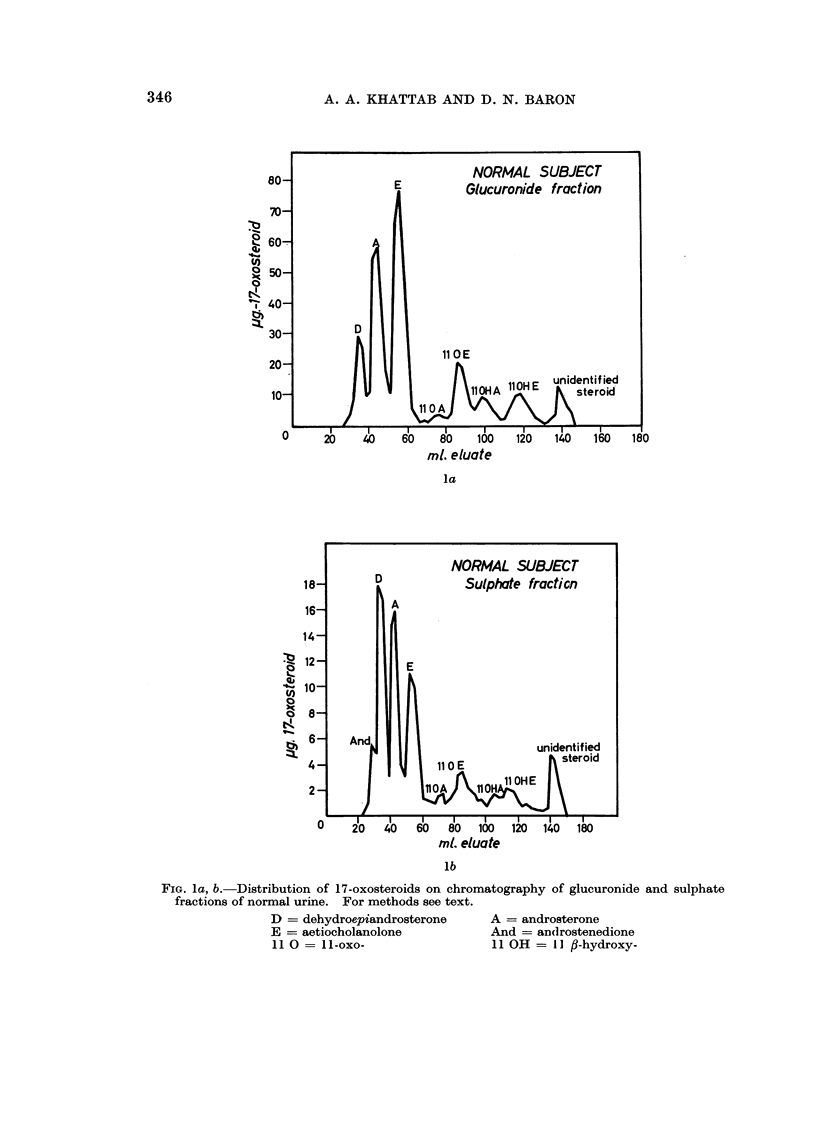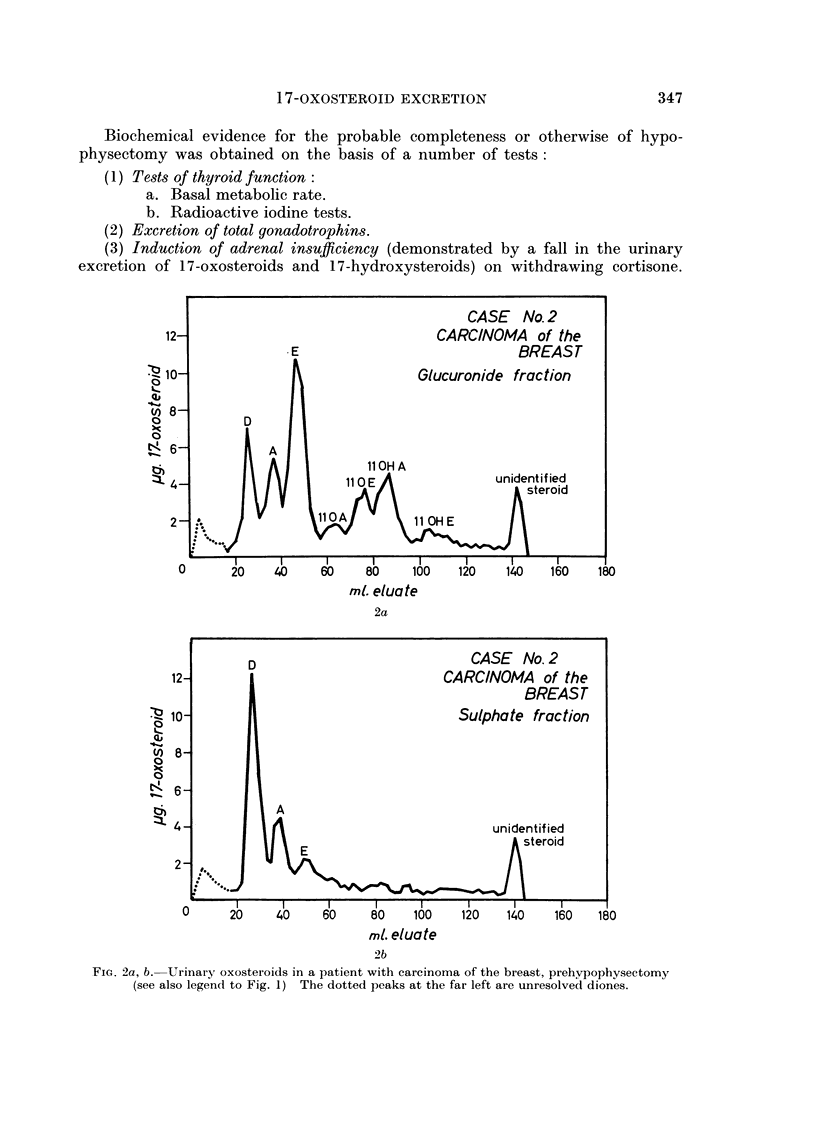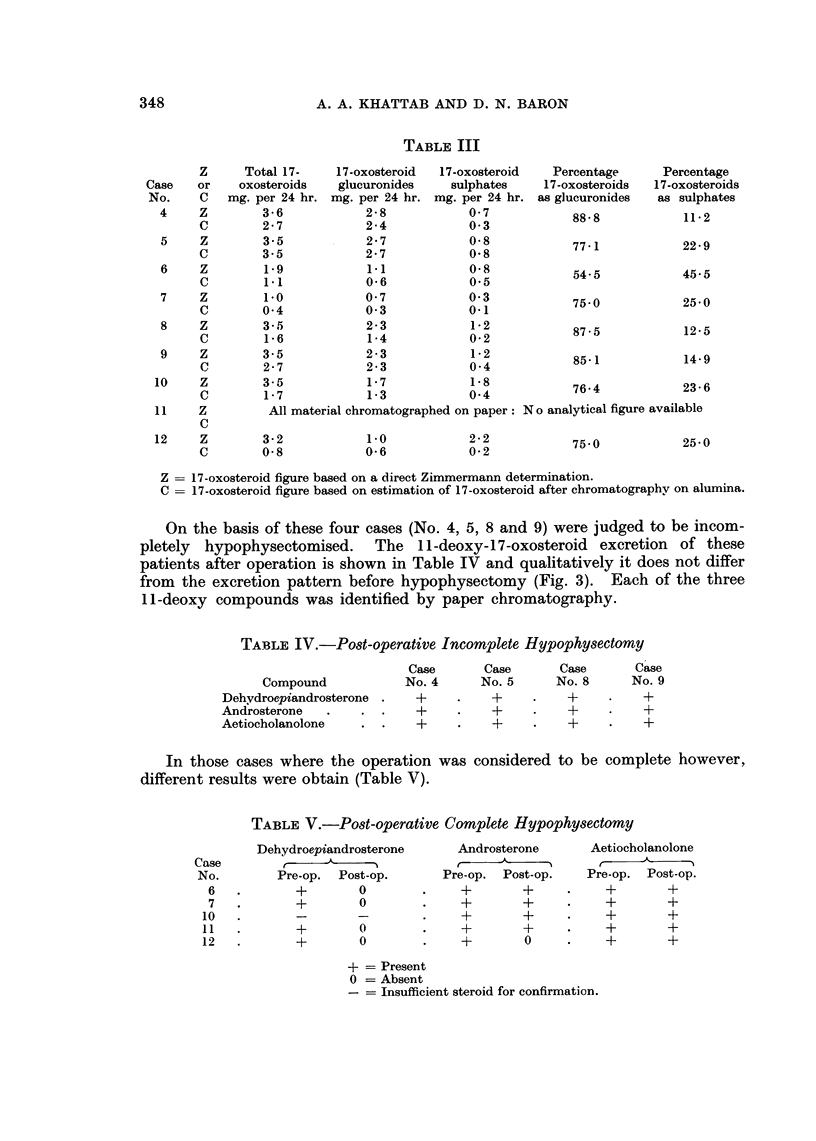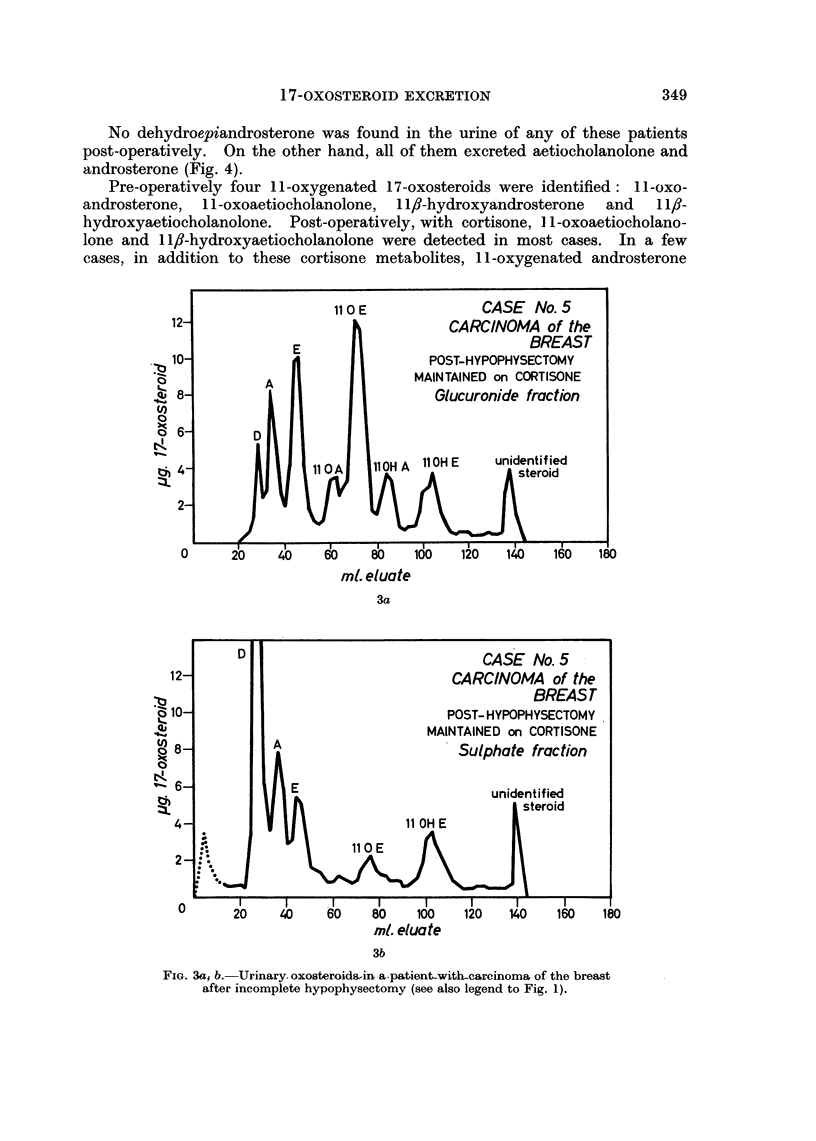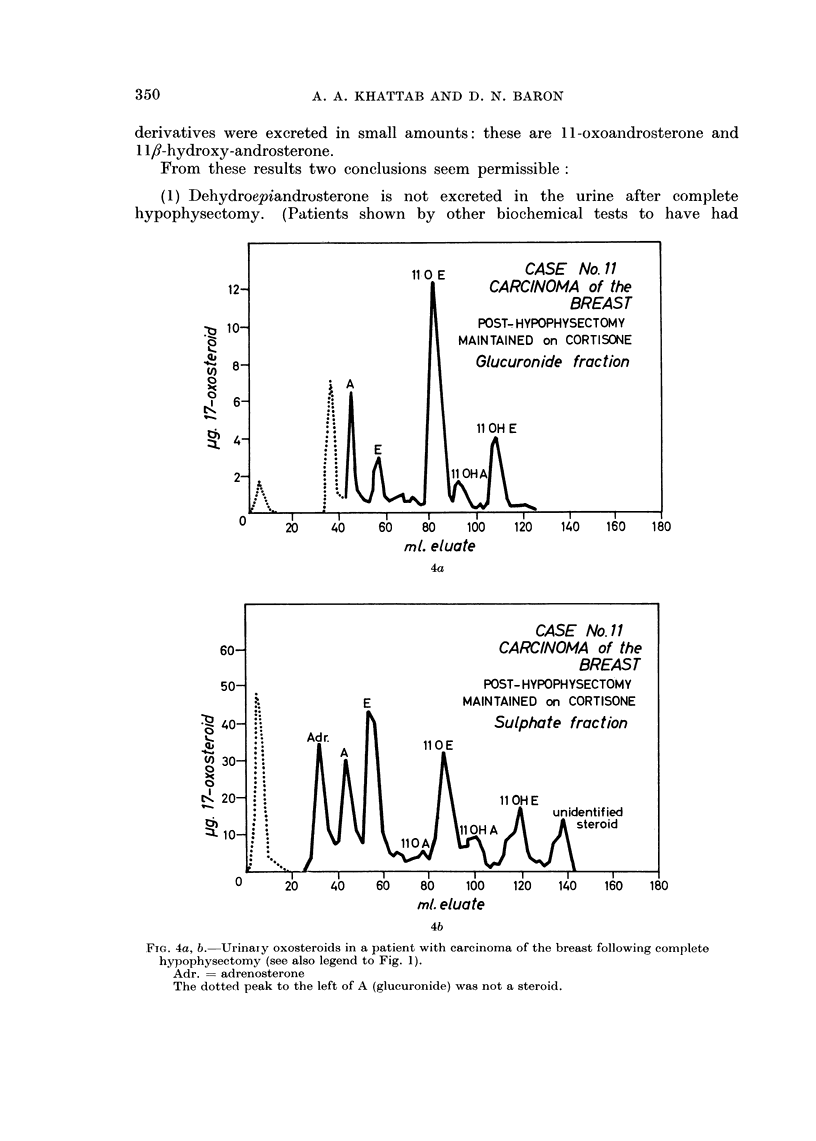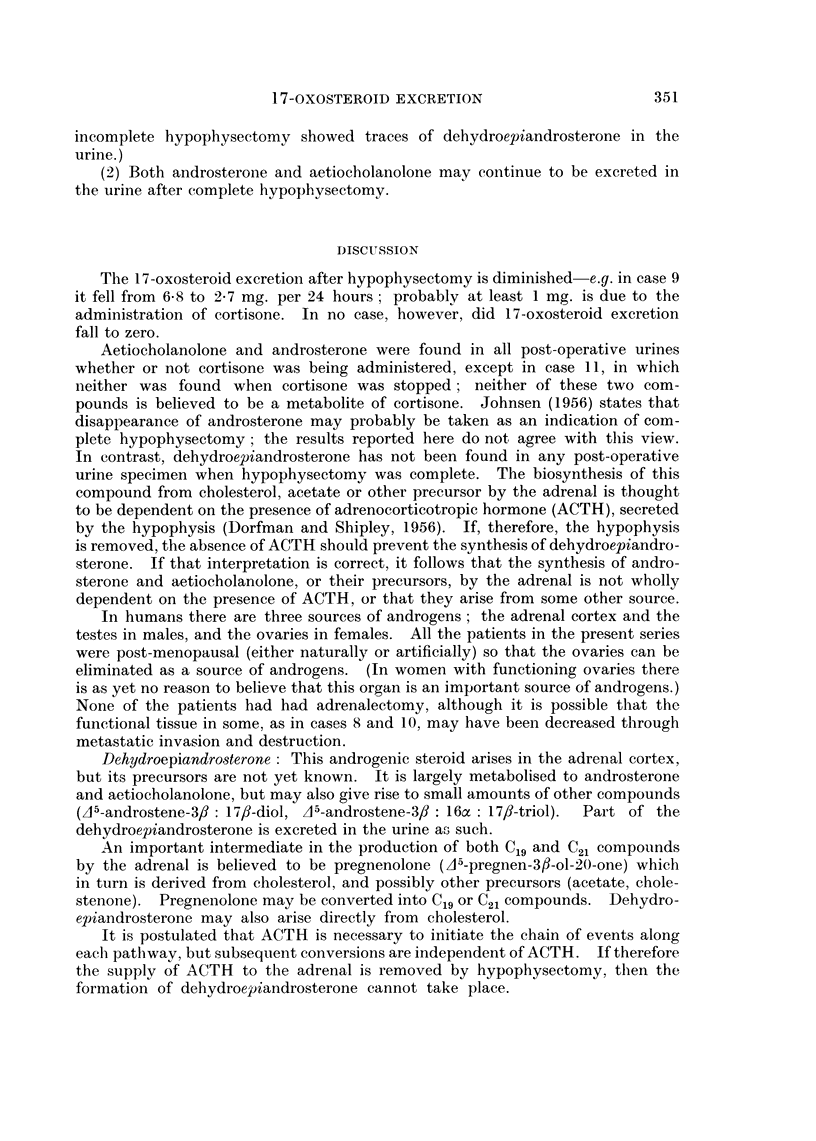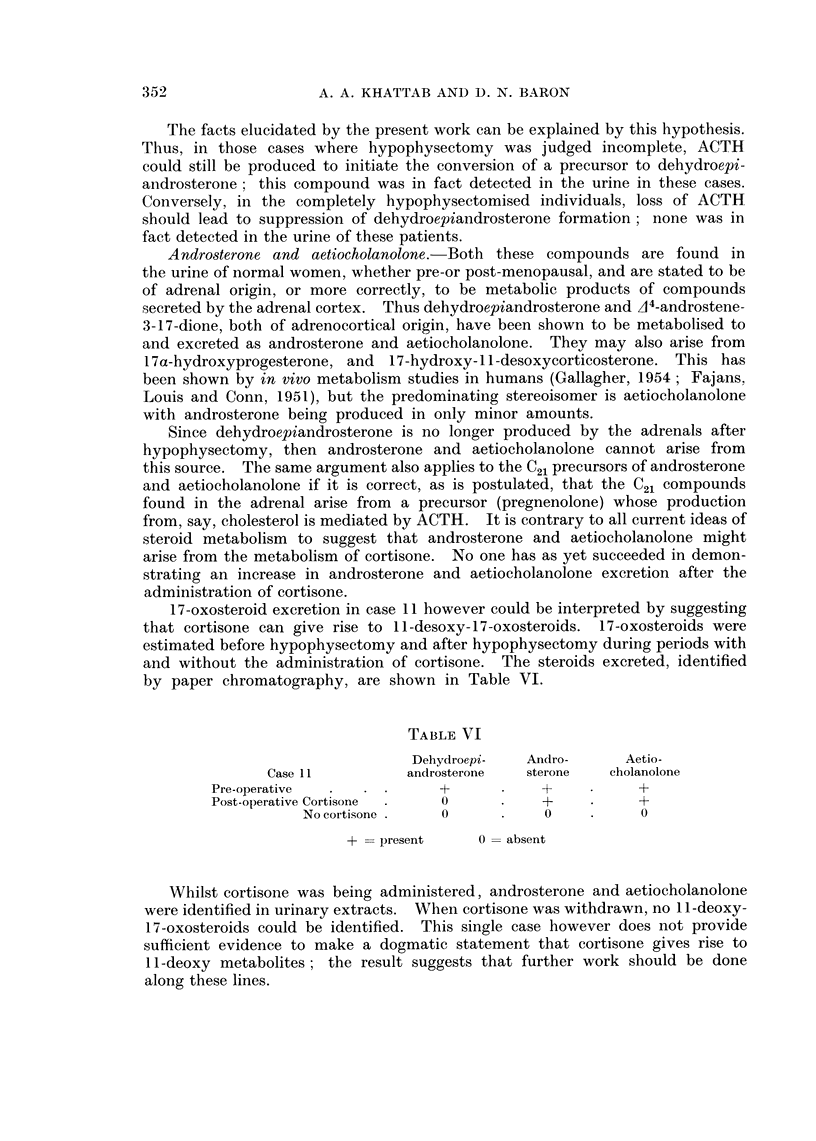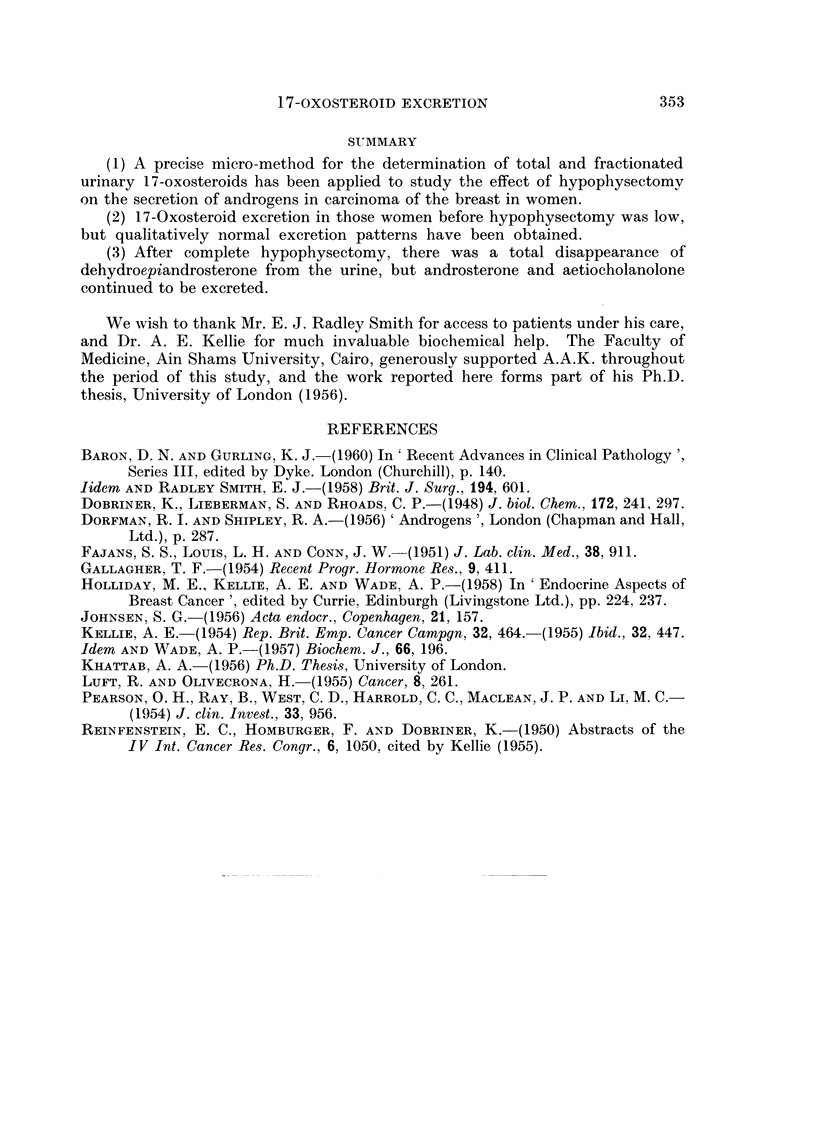# Differential 17-Oxosteroid Excretion in Women with Cancer of the Breast Following Hypophysectomy

**DOI:** 10.1038/bjc.1964.40

**Published:** 1964-06

**Authors:** A. A. Khattab, D. N. Baron


### DIFFERENTIAL 17-OXOSTEROID EXCRETION IN WOMEN WITH CANCER OF THE BREAST FOLLOWING HYPOPHYSECTOMY

A. A. KHATTAB* AND D. N. BARON

*From the Department of Chemical Pathology, The Royal Free Hospital, London, W.C.*1

Received for publication March 25, 1964

HYPOPHYSECTOMY was introduced for the treatment of cancer of the breast in women by Pearson, Ray, West, Harrold, MacLean and Li (1954) and by Luft and Olivecrona (1955). Only about 50 per cent of these cancers show a response, the so-called hormone-dependent tumours. With these tumours it has been found that a better response is obtained with complete removal of the pituitary than with incomplete hypophysectomy, as the former causes the greater modification of the hormone environment.

It is of concern in the management of patients submitted to hypophysectomy that a satisfactory means of assessing completeness of hypophysectomy be used. The methods used principally involve measurement of pituitary hormone or target organ hormone production, and gonadotrophins, adrenocortical function and thyroid function are commonly measured (Baron and Gurling, 1960).

After hypophysectomy, patients are maintained on cortisone. They excrete small quantities of 17-oxosteroids, which may be derived from the administered cortisone, or from the adrenal cortex under the stimulus of ACTH from remnants of incompletely removed pituitary. To investigate this it is necessary to perform differential analysis of the urinary 17-oxosteroids. The two 17-oxosteroid metabolites of cortisone which are 11-oxoaetiocholanolone and 11$\beta$-hydroxyaetiocholanolone, (3$\alpha$-hydroxy-5$\beta$-androstane-11 : 17-dione and 3$\alpha$ : 11$\beta$-dihydroxy-5$\beta$-androstane-17-one) are readily separated from the major $C_{11}$-deoxy-17-oxosteroids, dehydro*epi*androsterone, androsterone and aetiocholanolone, which are endogenous products.

Johnsen (1956) used this approach and published one excretion pattern of 17-oxosteroids from a patient with cancer of the breast, treated by hypophysectomy. He claimed that the entire disappearance of androsterone is a reliable sign of the completeness of hypophysectomy.

In the present work a method more refined than that used by Johnsen has been applied to the study of 17-oxosteroid excretion in cases of women with cancer of breast, to detect any change in their excretion pattern after hypophysectomy and to try to establish whether or not hypophysectomy has been complete.

This paper is an enlargement on research already given in a Ph.D. thesis (Khattab, 1956) and on the work of Holliday, Kellie and Wade (1958).

### MATERIAL AND METHODS

All the patients who underwent hypophysectomy were suffering from carcinoma of the breast with metastases. All but two cases were post-menopausal,

* Present address : Ain Shams Faculty of Medicine, Cairo, Egypt.

or had been ovariectomized. Of the twelve patients so far studied, one underwent pituitary stalk section (Case 7), the rest had surgical removal of the pituitary. After an initial period in hospital for investigation and assessment, surgical hypophysectomy was performed by the transfrontal approach and 7·5 mc of $^{198}$Au was inserted (as seeds) into the pituitary fossa (Baron, Gurling and Radley Smith, 1958). Cortisone was given, in variable doses depending on the patient's condition, to cover the operation and for maintenance. Thyroid replacement therapy was given post-operatively.

Pre-operative and post-operative studies were performed on the urinary 17-oxosteroid excretion when cortisone was being given, and when possible post-operatively and not receiving cortisone. The method adopted in this work was the one published by Kellie and Wade (1957) which consisted of (a) extraction of the steroid conjugates from the urine (which has no preservative), (b) hydrolysis of the glucuronide and sulphate fractions independently, (c) gradient elution chromatography on alumina, (d) estimation of individual steroids, (e) paper chromatography and infra-red spectroscopy for identification of steroids whenever possible.

### RESULTS

*Unique difficulties encountered during the processing of urines from patients with cancer of the breast*

In applying to urines from patients with cancer of the breast the method of 17-oxosteroid analysis already used satisfactorily with other urines, new problems were encountered which made the evaluation of the results before and after hypophysectomy very difficult. A primary cause of these problems was the low output of total 17-oxosteroids and (after hypophysectomy) the polyuria which made it necessary to process very large volumes of urine in order to obtain measurable amounts of 17-oxosteroid. Excessive amounts of a red pigment (presumably indigo red, Dobriner, Lieberman and Rhoads, 1948) present in many fractions made it extremely difficult to get an accurate measure of the 17-oxosteroid content of the urines or the chromatographic fractions. Absorption at 520 m$\mu$ in the Zimmermann reaction, which appeared to indicate 17-oxosteroid content, was shown to be falsely high when the material was chromatographed on paper for the purposes of identification. An example will illustrate these points.

*Case* 12.—Analysis of a pre-hypophysectomy urine gave a total 17-oxosteroid excretion of 4·2 mg. per 24 hours, 1·3 mg. as glucuronides and 2·9 mg. as sulphates. Chromatography of the 17-oxosteroids from the glucuronide fraction gave a final accuracy of 56·0 per cent, whilst from the 17-oxosteroids excreted as sulphates, the accuracy after chromatography on alumina was only 8 per cent. From these figures therefore the amount of steroid really excreted was 0·7 mg. as glucuronide and 0·23 mg. as sulphate, a total of less than 1·0 mg. per 24 hours.

In other cases, chromatography on alumina gave apparently well-defined peaks in recognisable positions, but paper chromatography failed to reveal the presence of the expected steroids. Such has been the case even when readings in the spectrophotometer showed maximum absorption at 520 m$\mu$ indicating, as one might think, the presence of a 17-oxosteroid.

It is apparent therefore that the neutral steroid extracts obtained from the urine of patients under discussion all contained pigment which produced, under the conditions of the Zimmermann reaction, the same colour as 17-oxosteroid. The application of Allen's correction which assumes a linear background to the spectrophotometer readings was not sufficient to eliminate this effect.

In general, however, on paper chromatograms the pigment had an $R_f$ value different from that of the 17-oxosteroids and did not interfere with the identification of compounds or the interpretation of the results.

From a quantitative point of view, therefore, the figures obtained for 17-oxosteroid excretion lack precision, and emphasis has been placed largely on the qualitative aspect of the results.

In older methods of steroid analysis separation of the neutral extract into ketonic and non-ketonic fractions, by means of Girard's reagent T, was a standard procedure, essential for satisfactory work. Milder methods of hydrolysis have, generally speaking, made this step unnecessary since less pigment is produced when hot acid hydrolysis is avoided.

In the present work on urines from hypophysectomised patients it was initially considered that this step was unnecessary and almost all of the preliminary work had been done before the difficulties inherent in the procedure were appreciated. More reliable quantitative figures could possibly have been obtained if the Girard separation had been applied to all urines instead of only to the very highly pigmented fractions.

In those instances where a Girard separation was performed, the recovery of 17-oxosteroids agreed very closely with the results after chromatography on alumina. For example the sulphate fraction from case 12, both pre- and post-operative, was subjected to a Girard separation, after removal of an aliquot for chromatography. The results are presented in Table I.

TABLE I

| Case 12 | 17-oxosteroid mg. per 24 hr. Before Girard separation | 17-oxosteroid mg. per 24 hr. After Girard separation | Percentage accuracy after Girard separation | Percentage accuracy after chromatography of material before Girard separation |
|---|---|---|---|---|
| Pre-op. | 2·9 | 0·23 | 8·0 | 8·0 |
| Post-op. | 2·2 | 0·17 | 8·0 | 7·7 |

In presenting the quantitative results therefore two figures have been given where possible.

(1) The 17-oxosteroid figure based on a direct Zimmermann determination.

(2) A corrected 17-oxosteroid figure based on the estimation of 17-oxosteroids after chromatography on alumina.

The pre-operative figures for the 17-oxosteroid excretion obtained in these cases of carcinoma of the breast are all very low, as found generally by other workers. The results are presented in Table II.

Apart from case 9, the daily excretion barely exceeds 1·0 mg. This is considerably below the lowest range of normal excretion. Most of the patients were suffering from widespread malignant disease. A further possible reason for low 17-oxosteroid excretion in patients with carcinoma of the breast is that they may have destructive secondaries in the adrenals. It is notable however that no matter how low the excretion both forms of conjugation are found; sometimes

TABLE II

| Case No. | Z or C | Total 17-oxosteroids mg. per 24 hr. | 17-oxosteroid glucuronides mg. per 24 hr. | 17-oxosteroid sulphates mg. per 24 hr. | Percentage 17-oxosteroids as glucuronides | Percentage 17-oxosteroids as sulphates |
|---|---|---|---|---|---|---|
| 1 | Z | 1·90 | 0·85 | 1·05 | 44·7 | 55·3 |
| | C | 1·05 | 0·47 | 0·58 | | |
| 2 | Z | 1·30 | 0·55 | 0·75 | 41·8 | 58·2 |
| | C | 0·86 | 0·36 | 0·50 | | |
| 3 | Z | 1·80 | 1·25 | 0·54 | 70·0 | 30·0 |
| | C | 0·87 | 0·60 | 0·27 | | |
| 9 | Z | 8·80 | 7·30 | 1·50 | 80·9 | 19·1 |
| | C | 6·80 | 5·50 | 1·30 | | |
| 10 | Z | 0·60 | 0·10 | 0·50 | 20·0 | 80·0 |
| | C | 0·20 | 0·04 | 0·16 | | |
| 11 | Z | 2·90 | 1·70 | 1·20 | 70·0 | 30·0 |
| | C | 1·70 | 1·20 | 0·50 | | |
| 12 | Z | 4·20 | 1·30 | 2·90 | 75·7 | 24·3 |
| | C | 0·95 | 0·72 | 0·23 | | |

Z = 17-oxosteroid figure based on a direct Zimmermann determination.
C = 17-oxosteroid figure based on estimation of 17-oxosteroid after chromatography on alumina.

the sulphate fraction predominates. In spite of the low 17-oxosteroid excretion, the chromatographic pattern of excretion remains substantially normal in so far as the types of compound excreted are concerned. Other workers (e.g. Kellie, 1954) have shown that all the compounds present in the urines of normal individuals (Fig. 1) may be excreted by patients with carcinoma of the breast. The claim that 11$\beta$-hydroxyaetiocholanolone, a minor component of the 17-oxosteroid fraction is significantly associated with neoplastic growth (Reifenstein, Homburger and Dobriner, 1950) has been disproved (Kellie, 1955); the compound has been found in the urine of all normal individuals examined. In the present series of cases, paper chromatography revealed the presence of all expected compounds pre-operatively. Of the 11-deoxy compounds, androsterone and aetiocholanolone were always present, dehydro*epi*androsterone was not detected in one case (No. 10); but the amount of material available for chromatography was very small.

Each of the four $C_{11}$-oxygenated 17-oxosteroids normally excreted was found in every patient studied. The 17-oxosteroid excretion of these carcinomatous patients does not differ quantitatively in any significant way from the results reported by other workers. Nor does it differ in its constituents from normal individuals which were analysed for comparative purposes (Fig. 2).

*Effect of hypophysectomy on 17-oxosteroid excretion*

Total 17-oxosteroid excretion after hypophysectomy is shown in Table III (Uncorrected and corrected figures).

The figures for 17-oxosteroid are very similar to those obtained pre-hypophysectomy. It must be remembered, however, that after hypophysectomy the patients were receiving a maintenance dose of cortisone; the excretion figures therefore do not represent metabolites only of endogenous hormone secretion. In all cases the presence of metabolites of cortisone was demonstrated by paper chromatography.

1*a*

1*b*

FIG. 1*a*, *b*.—Distribution of 17-oxosteroids on chromatography of glucuronide and sulphate fractions of normal urine. For methods see text.

D = dehydro*epi*androsterone  A = androsterone

E = aetiocholanolone  And = androstenedione

11 O = 11-oxo-  11 OH = 11 β-hydroxy-

Biochemical evidence for the probable completeness or otherwise of hypophysectomy was obtained on the basis of a number of tests :

(1) *Tests of thyroid function* :
  a. Basal metabolic rate.
  b. Radioactive iodine tests.

(2) *Excretion of total gonadotrophins.*

(3) *Induction of adrenal insufficiency* (demonstrated by a fall in the urinary excretion of 17-oxosteroids and 17-hydroxysteroids) on withdrawing cortisone.

2*a*

2*b*

FIG. 2*a*, *b*.—Urinary oxosteroids in a patient with carcinoma of the breast, prehypophysectomy (see also legend to Fig. 1) The dotted peaks at the far left are unresolved diones.

### Table III

| Case No. | Z or C | Total 17-oxosteroids mg. per 24 hr. | 17-oxosteroid glucuronides mg. per 24 hr. | 17-oxosteroid sulphates mg. per 24 hr. | Percentage 17-oxosteroids as glucuronides | Percentage 17-oxosteroids as sulphates |
|---|---|---|---|---|---|---|
| 4 | Z | 3·6 | 2·8 | 0·7 | 88·8 | 11·2 |
| | C | 2·7 | 2·4 | 0·3 | | |
| 5 | Z | 3·5 | 2·7 | 0·8 | 77·1 | 22·9 |
| | C | 3·5 | 2·7 | 0·8 | | |
| 6 | Z | 1·9 | 1·1 | 0·8 | 54·5 | 45·5 |
| | C | 1·1 | 0·6 | 0·5 | | |
| 7 | Z | 1·0 | 0·7 | 0·3 | 75·0 | 25·0 |
| | C | 0·4 | 0·3 | 0·1 | | |
| 8 | Z | 3·5 | 2·3 | 1·2 | 87·5 | 12·5 |
| | C | 1·6 | 1·4 | 0·2 | | |
| 9 | Z | 3·5 | 2·3 | 1·2 | 85·1 | 14·9 |
| | C | 2·7 | 2·3 | 0·4 | | |
| 10 | Z | 3·5 | 1·7 | 1·8 | 76·4 | 23·6 |
| | C | 1·7 | 1·3 | 0·4 | | |
| 11 | Z | All material chromatographed on paper : No analytical figure available | | | | |
| | C | | | | | |
| 12 | Z | 3·2 | 1·0 | 2·2 | 75·0 | 25·0 |
| | C | 0·8 | 0·6 | 0·2 | | |

Z = 17-oxosteroid figure based on a direct Zimmermann determination.
C = 17-oxosteroid figure based on estimation of 17-oxosteroid after chromatography on alumina.

On the basis of these four cases (No. 4, 5, 8 and 9) were judged to be incompletely hypophysectomised. The 11-deoxy-17-oxosteroid excretion of these patients after operation is shown in Table IV and qualitatively it does not differ from the excretion pattern before hypophysectomy (Fig. 3). Each of the three 11-deoxy compounds was identified by paper chromatography.

### Table IV.—*Post-operative Incomplete Hypophysectomy*

| Compound | Case No. 4 | Case No. 5 | Case No. 8 | Case No. 9 |
|---|---|---|---|---|
| Dehydro*epi*androsterone | + | + | + | + |
| Androsterone | + | + | + | + |
| Aetiocholanolone | + | + | + | + |

In those cases where the operation was considered to be complete however, different results were obtain (Table V).

### Table V.—*Post-operative Complete Hypophysectomy*

| Case No. | Dehydro*epi*androsterone Pre-op. | Dehydro*epi*androsterone Post-op. | Androsterone Pre-op. | Androsterone Post-op. | Aetiocholanolone Pre-op. | Aetiocholanolone Post-op. |
|---|---|---|---|---|---|---|
| 6 | + | 0 | + | + | + | + |
| 7 | + | 0 | + | + | + | + |
| 10 | — | — | + | + | + | + |
| 11 | + | 0 | + | + | + | + |
| 12 | + | 0 | + | 0 | + | + |

+ = Present
0 = Absent
— = Insufficient steroid for confirmation.

No dehydro*epi*androsterone was found in the urine of any of these patients post-operatively. On the other hand, all of them excreted aetiocholanolone and androsterone (Fig. 4).

Pre-operatively four 11-oxygenated 17-oxosteroids were identified: 11-oxo-androsterone, 11-oxoaetiocholanolone, 11$\beta$-hydroxyandrosterone and 11$\beta$-hydroxyaetiocholanolone. Post-operatively, with cortisone, 11-oxoaetiocholanolone and 11$\beta$-hydroxyaetiocholanolone were detected in most cases. In a few cases, in addition to these cortisone metabolites, 11-oxygenated androsterone

3*a*

3*b*

FIG. 3*a*, *b*.—Urinary oxosteroids in a patient with carcinoma of the breast after incomplete hypophysectomy (see also legend to Fig. 1).

derivatives were excreted in small amounts: these are 11-oxoandrosterone and 11$\beta$-hydroxy-androsterone.

From these results two conclusions seem permissible :

(1) Dehydro*epi*androsterone is not excreted in the urine after complete hypophysectomy. (Patients shown by other biochemical tests to have had

4*a*

4*b*

FIG. 4*a*, *b*.—Urinary oxosteroids in a patient with carcinoma of the breast following complete hypophysectomy (see also legend to Fig. 1).

Adr. = adrenosterone

The dotted peak to the left of A (glucuronide) was not a steroid.

incomplete hypophysectomy showed traces of dehydro*epi*androsterone in the urine.)

(2) Both androsterone and aetiocholanolone may continue to be excreted in the urine after complete hypophysectomy.

### DISCUSSION

The 17-oxosteroid excretion after hypophysectomy is diminished—*e.g.* in case 9 it fell from 6·8 to 2·7 mg. per 24 hours; probably at least 1 mg. is due to the administration of cortisone. In no case, however, did 17-oxosteroid excretion fall to zero.

Aetiocholanolone and androsterone were found in all post-operative urines whether or not cortisone was being administered, except in case 11, in which neither was found when cortisone was stopped; neither of these two compounds is believed to be a metabolite of cortisone. Johnsen (1956) states that disappearance of androsterone may probably be taken as an indication of complete hypophysectomy; the results reported here do not agree with this view. In contrast, dehydro*epi*androsterone has not been found in any post-operative urine specimen when hypophysectomy was complete. The biosynthesis of this compound from cholesterol, acetate or other precursor by the adrenal is thought to be dependent on the presence of adrenocorticotropic hormone (ACTH), secreted by the hypophysis (Dorfman and Shipley, 1956). If, therefore, the hypophysis is removed, the absence of ACTH should prevent the synthesis of dehydro*epi*androsterone. If that interpretation is correct, it follows that the synthesis of androsterone and aetiocholanolone, or their precursors, by the adrenal is not wholly dependent on the presence of ACTH, or that they arise from some other source.

In humans there are three sources of androgens; the adrenal cortex and the testes in males, and the ovaries in females. All the patients in the present series were post-menopausal (either naturally or artificially) so that the ovaries can be eliminated as a source of androgens. (In women with functioning ovaries there is as yet no reason to believe that this organ is an important source of androgens.) None of the patients had had adrenalectomy, although it is possible that the functional tissue in some, as in cases 8 and 10, may have been decreased through metastatic invasion and destruction.

*Dehydro*epi*androsterone*: This androgenic steroid arises in the adrenal cortex, but its precursors are not yet known. It is largely metabolised to androsterone and aetiocholanolone, but may also give rise to small amounts of other compounds ($\Delta^5$-androstene-3$\beta$ : 17$\beta$-diol, $\Delta^5$-androstene-3$\beta$ : 16$\alpha$ : 17$\beta$-triol). Part of the dehydro*epi*androsterone is excreted in the urine as such.

An important intermediate in the production of both $C_{19}$ and $C_{21}$ compounds by the adrenal is believed to be pregnenolone ($\Delta^5$-pregnen-3$\beta$-ol-20-one) which in turn is derived from cholesterol, and possibly other precursors (acetate, cholestenone). Pregnenolone may be converted into $C_{19}$ or $C_{21}$ compounds. Dehydro*epi*androsterone may also arise directly from cholesterol.

It is postulated that ACTH is necessary to initiate the chain of events along each pathway, but subsequent conversions are independent of ACTH. If therefore the supply of ACTH to the adrenal is removed by hypophysectomy, then the formation of dehydro*epi*androsterone cannot take place.

The facts elucidated by the present work can be explained by this hypothesis. Thus, in those cases where hypophysectomy was judged incomplete, ACTH could still be produced to initiate the conversion of a precursor to dehydro*epi*-androsterone ; this compound was in fact detected in the urine in these cases. Conversely, in the completely hypophysectomised individuals, loss of ACTH should lead to suppression of dehydro*epi*androsterone formation ; none was in fact detected in the urine of these patients.

*Androsterone and aetiocholanolone.*—Both these compounds are found in the urine of normal women, whether pre-or post-menopausal, and are stated to be of adrenal origin, or more correctly, to be metabolic products of compounds secreted by the adrenal cortex. Thus dehydro*epi*androsterone and $\Delta^4$-androstene-3-17-dione, both of adrenocortical origin, have been shown to be metabolised to and excreted as androsterone and aetiocholanolone. They may also arise from 17$\alpha$-hydroxyprogesterone, and 17-hydroxy-11-desoxycorticosterone. This has been shown by *in vivo* metabolism studies in humans (Gallagher, 1954 ; Fajans, Louis and Conn, 1951), but the predominating stereoisomer is aetiocholanolone with androsterone being produced in only minor amounts.

Since dehydro*epi*androsterone is no longer produced by the adrenals after hypophysectomy, then androsterone and aetiocholanolone cannot arise from this source. The same argument also applies to the $C_{21}$ precursors of androsterone and aetiocholanolone if it is correct, as is postulated, that the $C_{21}$ compounds found in the adrenal arise from a precursor (pregnenolone) whose production from, say, cholesterol is mediated by ACTH. It is contrary to all current ideas of steroid metabolism to suggest that androsterone and aetiocholanolone might arise from the metabolism of cortisone. No one has as yet succeeded in demonstrating an increase in androsterone and aetiocholanolone excretion after the administration of cortisone.

17-oxosteroid excretion in case 11 however could be interpreted by suggesting that cortisone can give rise to 11-desoxy-17-oxosteroids. 17-oxosteroids were estimated before hypophysectomy and after hypophysectomy during periods with and without the administration of cortisone. The steroids excreted, identified by paper chromatography, are shown in Table VI.

TABLE VI

| Case 11 | | Dehydro*epi*-androsterone | Andro-sterone | Aetio-cholanolone |
|---|---|---|---|---|
| Pre-operative | | + | + | + |
| Post-operative | Cortisone | 0 | + | + |
| | No cortisone | 0 | 0 | 0 |

+ = present 0 = absent

Whilst cortisone was being administered, androsterone and aetiocholanolone were identified in urinary extracts. When cortisone was withdrawn, no 11-deoxy-17-oxosteroids could be identified. This single case however does not provide sufficient evidence to make a dogmatic statement that cortisone gives rise to 11-deoxy metabolites ; the result suggests that further work should be done along these lines.

SUMMARY

(1) A precise micro-method for the determination of total and fractionated urinary 17-oxosteroids has been applied to study the effect of hypophysectomy on the secretion of androgens in carcinoma of the breast in women.

(2) 17-Oxosteroid excretion in those women before hypophysectomy was low, but qualitatively normal excretion patterns have been obtained.

(3) After complete hypophysectomy, there was a total disappearance of dehydro*epi*androsterone from the urine, but androsterone and aetiocholanolone continued to be excreted.

We wish to thank Mr. E. J. Radley Smith for access to patients under his care, and Dr. A. E. Kellie for much invaluable biochemical help. The Faculty of Medicine, Ain Shams University, Cairo, generously supported A.A.K. throughout the period of this study, and the work reported here forms part of his Ph.D. thesis, University of London (1956).

REFERENCES

BARON, D. N. AND GURLING, K. J.—(1960) In ' Recent Advances in Clinical Pathology ', Series III, edited by Dyke. London (Churchill), p. 140.

*Iidem* AND RADLEY SMITH, E. J.—(1958) *Brit. J. Surg.*, **194**, 601.

DOBRINER, K., LIEBERMAN, S. AND RHOADS, C. P.—(1948) *J. biol. Chem.*, **172**, 241, 297.

DORFMAN, R. I. AND SHIPLEY, R. A.—(1956) ' Androgens ', London (Chapman and Hall, Ltd.), p. 287.

FAJANS, S. S., LOUIS, L. H. AND CONN, J. W.—(1951) *J. Lab. clin. Med.*, **38**, 911.

GALLAGHER, T. F.—(1954) *Recent Progr. Hormone Res.*, **9**, 411.

HOLLIDAY, M. E., KELLIE, A. E. AND WADE, A. P.—(1958) In ' Endocrine Aspects of Breast Cancer ', edited by Currie, Edinburgh (Livingstone Ltd.), pp. 224, 237.

JOHNSEN, S. G.—(1956) *Acta endocr., Copenhagen*, **21**, 157.

KELLIE, A. E.—(1954) *Rep. Brit. Emp. Cancer Campgn*, **32**, 464.—(1955) *Ibid.*, **32**, 447.

*Idem* AND WADE, A. P.—(1957) *Biochem. J.*, **66**, 196.

KHATTAB, A. A.—(1956) *Ph.D. Thesis*, University of London.

LUFT, R. AND OLIVECRONA, H.—(1955) *Cancer*, **8**, 261.

PEARSON, O. H., RAY, B., WEST, C. D., HARROLD, C. C., MACLEAN, J. P. AND LI, M. C.—(1954) *J. clin. Invest.*, **33**, 956.

REINFENSTEIN, E. C., HOMBURGER, F. AND DOBRINER, K.—(1950) Abstracts of the *IV Int. Cancer Res. Congr.*, **6**, 1050, cited by Kellie (1955).